# HTRA1-dependent proteolysis induces age-related retinal degeneration and exacerbates choroidal neovascularization

**DOI:** 10.1242/dmm.052253

**Published:** 2025-09-17

**Authors:** Kenneth J. Katschke, Tom Truong, Victoria Pham, Hongkang Xi, Wanjian Tang, Xiaowu Gu, Pooja Teotia, Jeffrey W. Hofmann, Shawnta Y. Chaney, Daniel Kirchhofer, Menno van Lookeren Campagne, Marion Jeanne

**Affiliations:** ^1^ Department of Immunology, Genentech Inc., South San Francisco, CA 94080, USA; ^2^ Department of Translational Immunology, Genentech Inc., South San Francisco, CA 94080, USA; ^3^ Department of Microchemistry, Proteomics & Lipidomics, Genentech Inc., South San Francisco, CA 94080, USA; ^4^ Department of Early Discovery Biochemistry, Genentech Inc., South San Francisco, CA 94080, USA; ^5^ Department of Neuroscience, Genentech Inc., South San Francisco, CA 94080, USA; ^6^ Department of Research Pathology, Genentech Inc., South San Francisco, CA 94080, USA

**Keywords:** HTRA1, Age-related macular degeneration, Retinal degeneration, Choroidal neovascularization, RBP3

## Abstract

Polymorphisms in the *ARMS2/HTRA1* locus on chromosome 10 enhance the risk of geographic atrophy and macular neovascularization, the advanced forms of age-related macular degeneration (AMD). Although HTRA1 mutations have been associated with microvascular defects in the brain, it remains unclear whether changes in HTRA1 expression contribute to AMD pathophysiology. In this study, we showed that, in AMD donor eyes, HTRA1 protein accumulated around the retinal pigment epithelium (RPE)/photoreceptor lesions. We then demonstrated that overexpression of catalytically active, but not catalytically inactive, HTRA1 in RPE cells in mice induced age-dependent loss of photoreceptors, inflammation and a decline in photoreceptor functional responses. This retinal degeneration was not exacerbated when the mice were exposed to phototoxic stress in the constant light exposure preclinical model. However, mice overexpressing catalytically active HTRA1 had significant exacerbation of laser-induced choroidal neovascularization lesions. Finally, as substrate processing may define the molecular basis for HTRA1-induced retinal degeneration, we initiated a proteomics approach and identified the visual cycle key player RBP3 as a disease-relevant HTRA1 substrate in the retina.

## Teaser

**Summary:** HTRA1 accumulates in retinas of patients with age-related macular degeneration. Its overexpression in mice causes age-related photoreceptor degeneration and inflammation and exacerbates neovascularization. These phenotypes depend on HTRA1 catalytic activity and may involve RBP3.

## INTRODUCTION

Age-related macular degeneration (AMD) is a leading cause of severe central vision loss in people aged 50 or older in higher-income countries (Flaxman et al., 2017). A characteristic feature of AMD pathology is the accumulation of extracellular basal laminar and basal linear deposits (i.e. soft drusen) within the macula between the retinal pigment epithelium (RPE) and Bruch's membrane. In advanced stages, AMD can progress to geographic atrophy (GA) and/or macular neovascularization (MNV). GA is defined by the loss of RPE and photoreceptors, whereas MNV is characterized by the growth of leaky blood vessels from the choroid across Bruch's membrane into the sub-RPE or subretinal space, or from the retinal circulation towards the outer retina. Both GA and MNV cause severe vision loss and often occur simultaneously (Holz et al., 2014; Pugazhendhi et al., 2021; Spaide et al., 2020). Although the etiology of AMD remains unclear, it is established that it is a multifactorial disease, with complex contributions from aging, genetic predisposition and environmental factors (Guymer and Campbell, 2023). Chronic inflammation, compromised extracellular matrix maintenance, and metabolic and oxidative stress have all been implicated in AMD pathophysiology; however, the underlying biological sequence of events that leads to advanced AMD remains largely unknown (Fleckenstein et al., 2021).

Human genetic analyses have greatly contributed to better understanding of the disease mechanisms underlying AMD risk and progression (DeWan et al., 2006; Edwards et al., 2005; Fritsche et al., 2016; Hageman et al., 2005; Haines et al., 2005; Klein et al., 2005; Winkler et al., 2020; Yan et al., 2018). Although AMD is a polygenic disease with many loci associated with modest contributions to the disease, two loci on chromosomes 1 and 10 account for more than 50% of AMD heritability (Fritsche et al., 2016). The identification of complement factor H (*CFH*) as the AMD-relevant gene at the 1q31 locus revealed the involvement of the complement system in the disease biology (Edwards et al., 2005; Hageman et al., 2005; Haines et al., 2005; Klein et al., 2005). The contribution of the chromosomal region 10q26 to AMD remains elusive and is controversial (Gogna et al., 2023; May et al., 2021; Merle et al., 2022). Two genes lie in close proximity at this locus, age-related maculopathy susceptibility 2 (*ARMS2*) and high temperature requirement A1 [*HTRA1*; Online Mendelian Inheritance in Man (OMIM) #602194]. Numerous AMD-associated variants identified in this region are almost in complete linkage disequilibrium, making it challenging to pinpoint the causative variant (Wang, 2014). Conflicting outcomes from extensive functional analysis make it difficult to know whether *ARMS2* or *HTRA1* is the AMD-relevant gene at this locus, and we cannot exclude the possibility that both genes are associated with AMD risk, or that neither of them is the causal gene.

HTRA1 is a secreted homotrimeric serine protease that is ubiquitously expressed in human tissues and has been implicated in a range of pathological conditions, such as cancer, preeclampsia, arthritic disease and cerebrovascular diseases (Eigenbrot et al., 2012; Grau et al., 2006; Hara et al., 2009; De Luca et al., 2003; Oka et al., 2022; Teoh et al., 2015; Uemura et al., 2020; Wang and Nie, 2021; Zurawa-Janicka et al., 2010, 2017). HTRA1 promotes TGFβ signaling by cleaving pro- or latent-TGFβ and contributes to nervous and vascular dysfunction (Beaufort et al., 2014; Friedrich et al., 2015; Jin et al., 2018; Launay et al., 2008; Li and Zhang, 2015; Liu et al., 2015; Oka et al., 2004; Shiga et al., 2011). Human genetics studies identified the loss of HTRA1 proteolytic function as causative of cerebral autosomal recessive arteriopathy with subcortical infarcts and leukoencephalopathy (OMIM #600142), and autosomal dominant mutations in the protease domain of HTRA1 were also linked to late-onset cerebral small vessel disease (Fukutake, 2011; Uemura et al., 2020; Verdura et al., 2015). Furthermore, the extracellular protease activity of HTRA1 is associated with bone mineralization (Canfield et al., 2007; Tiaden et al., 2012) and placental development (Hasan et al., 2015). In preclinical models of osteoarthritis, overexpression of HTRA1, but not the proteolytically inactive form [i.e. S328A (Hu et al., 1998)], degrades the cartilage by digesting its major components including aggrecan (Chamberland et al., 2009; Chen et al., 2019; Tsuchiya et al., 2005).

Overexpression of human HTRA1 by the RPE cells in the mouse eye has been shown to cause polypoidal choroidal vasculopathy (PCV), destabilization of Bruch's membrane with fragmentation of the elastic laminae and increased risk of choroidal neovascularization (CNV) (Jones et al., 2011; Kumar et al., 2014; Vierkotten et al., 2011; Navneet et al., 2024). Similarly, overexpression of HTRA1 using a ubiquitous promoter has been shown to increase angiogenic potential in mouse retina (Ahamed et al., 2022; Nakayama et al., 2014). On the contrary, a mild decrease in vascular density in the retina was reported in *Htra1* knockout mice (Zhang et al., 2012). Taken together, these studies support a role for HTRA1 in neovascularization in patients with AMD. Of note, variability in the vascular phenotypes in *HTRA1* transgenic and knockout mice has been observed between different studies, which could be attributed to the transgenic strategy, mouse genetic background and environmental factors that would influence the penetrance and severity of the phenotypes (Ahamed et al., 2022; Jones et al., 2011; Kumar et al., 2014; Nakayama et al., 2014; Vierkotten et al., 2011). A lack of consensus is also true for non-vascular retinal phenotypes observed in *HTRA1*-overexpressing transgenic animals: some studies described RPE atrophy and photoreceptor degeneration, whereas others reported no phenotypes (Ahamed et al., 2022; Jones et al., 2011; Nakayama et al., 2014; Oura et al., 2018; Vierkotten et al., 2011).

The unclear effect of the *ARMS2/HTRA1* genetic locus on AMD pathophysiology and the controversial therapeutic approach proposed to help patients (i.e. inhibition or overexpression of HTRA1) (Csaky et al., 2022; Merle et al., 2022; Pan et al., 2022; Williams et al., 2021) led us to investigate HTRA1 activity in retinal homeostasis and in response to AMD-relevant stressors. Because we observed more HTRA1 protein in retinal lesions from patients with AMD than in those from unaffected controls, we specifically assessed whether enhanced HTRA1 activity could confer therapeutic benefits or be harmful to the retina. We overexpressed catalytically active and inactive forms of HTRA1 in RPE cells of mice to test potential effect on photoreceptor or RPE survival. We also tested whether active HTRA1 could exacerbate the disease phenotypes in preclinical AMD models and performed a screen to identify disease-relevant HTRA1 substrates in the retina.

## RESULTS

### HTRA1 protein is expressed in the human eye and accumulates in AMD lesions

**Fig. 1.**
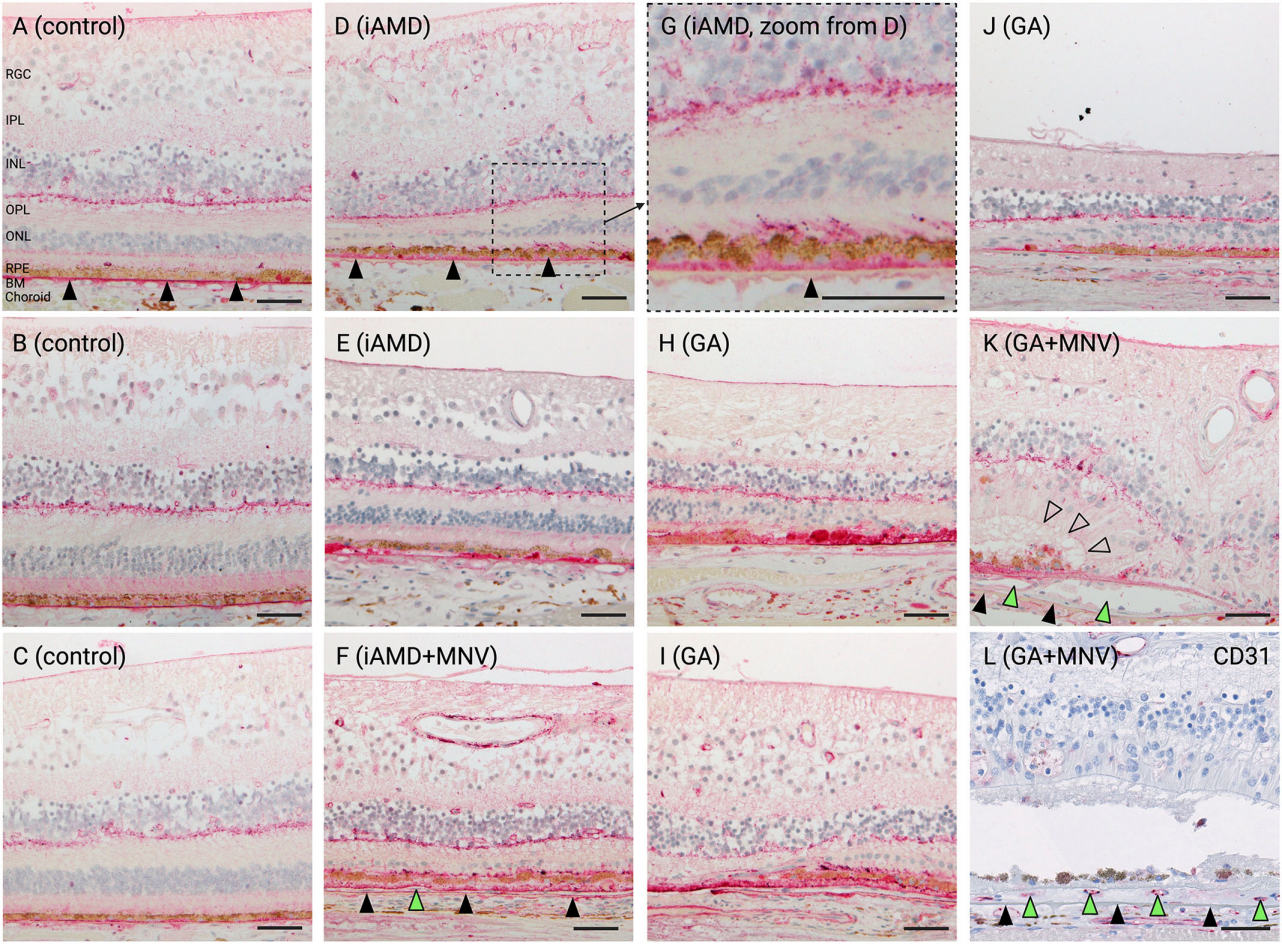
**HTRA1 protein accumulates in lesions present in human donor eyes with age-related macular degeneration (AMD).** Immunohistochemistry (IHC) showing HTRA1 distribution in retinas from unaffected controls, and patients with intermediate AMD (iAMD) and geographic atrophy (GA). Some retinas present macular neovascularization (MNV). (A-C) In control retinas, HTRA1 predominantly localizes to BM, the outer plexiform layer (excluding the Henle fiber layer) and horizontal cells, with variable, weaker staining of the photoreceptor layer. (D-G) Intermediate AMD retinas show HTRA1-positive basal laminar deposits, basal linear deposits and hard drusen between the RPE and BM, and accentuation of HTRA1 is seen at the RPE/photoreceptor interface, demonstrated in the magnification of D (G). (H-K) GA retinas show strong HTRA1 positivity in heavily pigmented cells associated with the degenerating RPE layer, which may represent displaced RPE cells or macrophages. (L) Parallel slide to K, showing CD31 IHC to confirm neovascularization. Filled arrowheads, BM; empty arrowheads, external limiting membrane descent; green arrowheads, neovascularization (vessels between BM and RPE). Each image is from a unique patient, except F and I, which are different eyes from the same patient, and K and L, which are from parallel sections from the same eye of a patient. Scale bars: 50 µm. RGC, retinal ganglion cell layer; IPL, inner plexiform layer; INL, inner nuclear layer; OPL, outer plexiform layer; ONL, outer nuclear layer; RPE, retinal pigment epithelium; BM, Bruch's membrane.

Because the mechanism by which the *ARMS2/HTRA1* genetic locus influences AMD risk remains controversial, and mRNA expression level does not always correlate with protein expression levels, we decided to assess HTRA1 protein expression in human eyes, independently of the donor genetic status (*ARMS2/HTRA1* risk allele carrier or not). We instead focused on disease status, to determine whether HTRA1 protein was more or less present in AMD lesions. HTRA1 protein expression was assessed using a monoclonal antibody that detects human HTRA1 in fixed paraffin-embedded human donor eyes. In healthy retinas, HTRA1 protein was mainly localized to Bruch's membrane and horizontal cells (Fig. 1A-C). Variable and weaker HTRA1 protein expression was also observed in the photoreceptor layer of control donors (Fig. 1A-C). In intermediate AMD (iAMD) eyes, HTRA1-positive basal laminar deposits, basal linear deposits and hard drusen between the RPE and Bruch's membrane were observed (Fig. 1D-F). There were also foci of increased HTRA1 protein signal in the photoreceptor outer segments (Fig. 1G). In more advanced AMD donor eyes with GA lesions, there were often large, heavily pigmented cells that were strongly immunoreactive for HTRA1 associated with the degenerating RPE layer (Fig. 1H-K). These cells may be displaced RPE cells or macrophages that engulfed dying RPE cell debris. Of note, this intensified HTRA1 protein presence in lesions was less present in GA lesions in which RPE cells were missing (Fig. 1J,K) and was absent in fibrous scar tissue of end-stage GA (Fig. S1A). We also confirmed MNV in a subset of samples (Fig. 1, green arrowheads) using anti-CD31 immunohistochemistry (IHC) (Fig. 1L). However, neovascular small vessels were negative for HTRA1, and, compared to AMD samples without neovascularization, samples with MNV did not show a different HTRA1 expression pattern.

In conclusion, HTRA1 protein is predominantly detected in Bruch's membrane and horizontal cells of unaffected control retinas. As AMD initiates, HTRA1 protein starts to accumulate in the extracellular matrix surrounding the RPE, with greater accumulation associated with degenerating RPE cells as the disease progresses to GA.

### HTRA1 overexpression leads to age-related retinal degeneration and loss of photoreceptor function

**Fig. 2.**
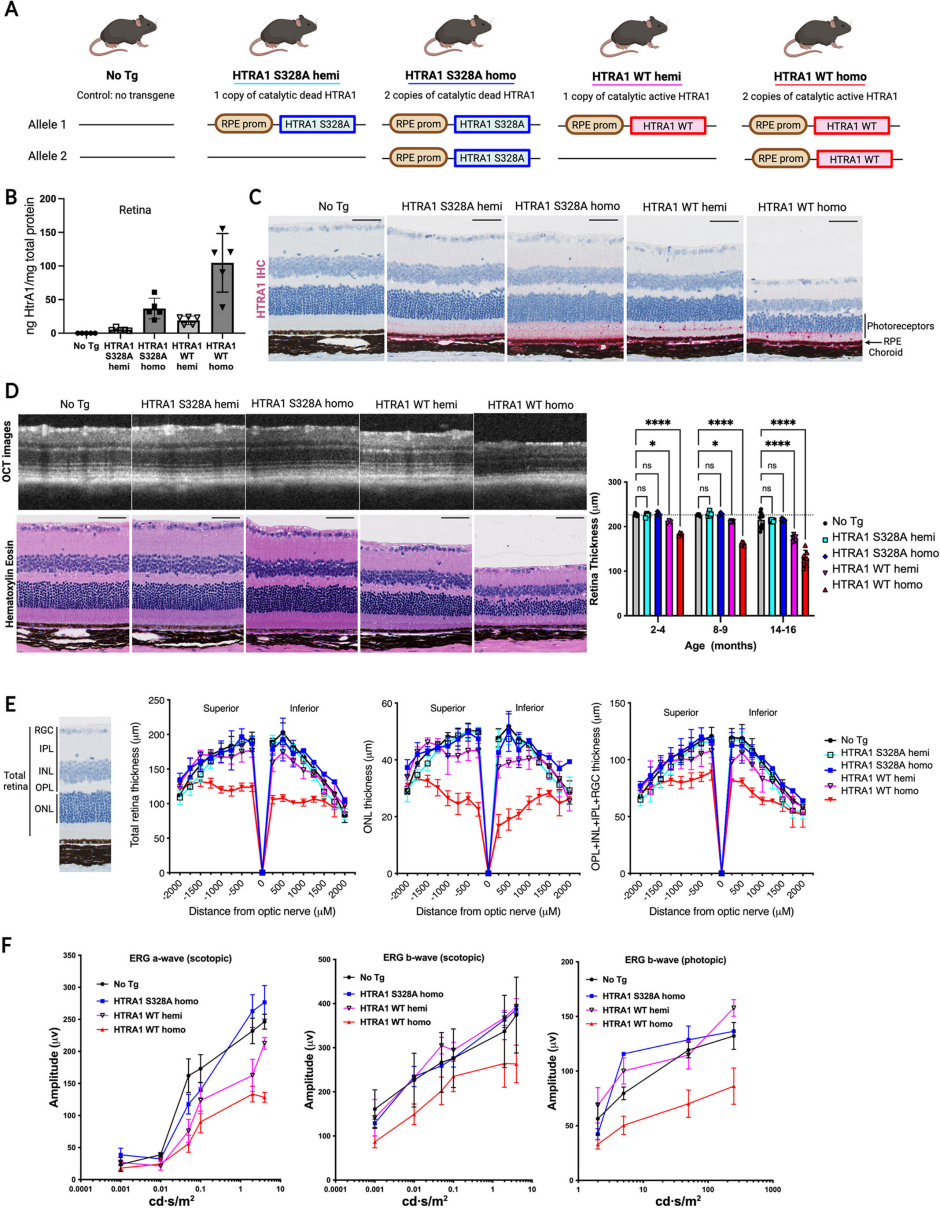
**Human HTRA1 overexpression in mice leads to age-related retinal degeneration and loss of photoreceptor function.** (A) Generation of transgenic mice expressing one (hemi, hemizygotes) or two copies (homo, homozygotes) of the human wild-type (WT) or catalytically inactive (S328A) HTRA1 under a RPE-specific promoter. Created in BioRender by Gu, X. (2025). https://biorender.com/kuqqr85. This figure was sublicensed under CC-BY 4.0 terms. (B) Human HTRA1 protein levels in the retina of the different mouse strains, normalized to the total protein amounts and detected using an enzyme-linked immunosorbent assay. *n*=5 per genotype. (C) IHC showing human HTRA1 protein in the RPE and photoreceptor segments of HTRA1 transgenic mice, but absent in the non-transgenic mice (No Tg). Representative images from a cohort of 11-month-old mice, *n*=5 to 6 per genotype. Scale bars: 50 µm. (D) Representative optical coherence tomography (OCT) scans (left, top row) and Hematoxylin and Eosin histology photomicrographs (left, bottom row) showing retinal thickness of the different mouse strains. Quantification of total retinal thickness by OCT, averaged across the entire retina (except 100 µm around the optic nerve head) over time (right), showing protease-dependent age-related degeneration in mice expressing HTRA1 WT (hemi and homo). Cohort of 9-month-old mice for the OCT scans and 11-month-old for the histology analysis, *n*=5 to 6 per genotype. Two-way ANOVA; ns, not significant; *P<0.05, ****P<0.0001. Scale bars: 50 µm. (E) Representative image (from C, No Tg) showing the different retinal layers. Morphometry analysis of the total retina, the outer nuclear layer (ONL) and the rest of the retina (OPL, outer plexiform layer; INL, inner nuclear layer; IPL, inner plexiform layer; RGC, retinal ganglion cell layer) of the different mouse strains at 11 months, showing protease-dependent pan-retinal thinning in mice expressing HTRA1 WT (hemi and homo). The decreased retinal thickness when measured by histology in comparison to OCT is expected and attributable to tissue contraction during processing. *n*=3 to 5 per genotype. (F) Electroretinogram measurements in the different mouse strains at 9 months showing protease-dependent photoreceptor function loss in mice expressing HTRA1 WT (hemi and homo). *n*=3 or 4 per genotype.

To determine whether increased HTRA1 expression is beneficial, deleterious or non-contributory to retinal health, we generated a transgenic mouse in which the human *HTRA1* gene was placed under the control of the RPE-specific *VMD2* promoter (Esumi et al., 2004). Furthermore, to determine whether HTRA1 enzymatic activity is responsible for any effect, we generated a similar transgenic mouse but replaced catalytically active HTRA1 [wild type (WT)] with catalytically inactive HTRA1 [S328A (Hu et al., 1998)]. Mice not carrying the transgene (non-transgenic), or carrying one transgene copy (hemizygous) or two transgene copies (homozygous) were used for experiments (Fig. 2A). Expression levels of the human *HTRA1* transgene in retinal lysates and RPE/choroid lysates obtained from WT or S328A versions of HTRA1 were comparable (Fig. 2B; Fig. S1B). IHC analysis using a human-specific anti-HTRA1 antibody confirmed that human HTRA1 protein was absent in the non-transgenic mice and was produced in the RPE cells of the HTRA1 WT and S328A transgenic animals (Fig. 2C). Interestingly, the analysis also revealed that HTRA1 accumulated in the photoreceptor inner and outer segment layer, localized between the outer nuclear layer (ONL) and the RPE (Fig. 2C).

Mice from the different colonies were aged, and ocular examination was performed. No obvious abnormalities were observed by fundus photography or fluorescent angiography in any of the mouse strains (Fig. S1C). Quantification of retinal blood vessel patterning also did not reveal any abnormalities, suggesting that HTRA1 overexpression did not cause any anatomic vascular defect (Fig. S1D). However, assessment of the retinal layer thickness by optical coherence tomography (OCT) scanning and histology revealed that HTRA1 overexpression by the RPE cells caused retinal degeneration, which was dependent on transgene dosage and age of the animals (Fig. 2D; Fig. S1E). As early as 2-4 months of age, HTRA1 WT hemizygous mice had a 16 µm reduction in retinal thickness (−7% compared to that in non-transgenic mice), and HTRA1 WT homozygous mice showed a 43 µm loss (−19% compared to that in non-transgenic mice). The retinal degeneration reached −42 µm (−20%) and −86 µm (−40%), respectively, by 14-16 months. In contrast, HTRA1 S328A transgenic mice showed normal retinal thickness, even at 14-16 months of age (hemizygous, 213±2 µm; homozygous, 215±2 µm; compared to 215±17 µm for the non-transgenic mice). Thus, our data support that the age- and dosage-dependent retinal degeneration is caused by HTRA1 protease activity. Next, we performed a quantitative morphometric assessment of retinal thickness, as well as of specific retinal layers, along the distance superiorly and inferiorly from the optic nerve head in the different mouse strains at 6 and 11 months. This analysis confirmed that HTRA1 WT overexpression caused age-related retinal degeneration across the entire retina (Fig. S1F, 6 months; Fig. 2E, 11 months), and further showed that the loss is largely due to photoreceptor degeneration (Fig. 2E; photoreceptor loss in the ONL, compared to loss in the rest of the retina). In line with our morphometric analysis, electroretinogram (ERG) measurements in the different mouse strains at 9 months found a significant reduction in the amplitude of photoreceptor-originated scotopic a-wave, but not bipolar cell-generated b-wave, in HTRA1 WT transgenic mice (Fig. 2F).

In conclusion, our data show that HTRA1 WT mice develop spontaneous age-related photoreceptor degeneration and functional loss, and this effect is dependent on *HTRA1* WT gene dosage as well as HTRA1 catalytic activity.

### HTRA1 overexpression leads to reactive retinal gliosis

**Fig. 3.**
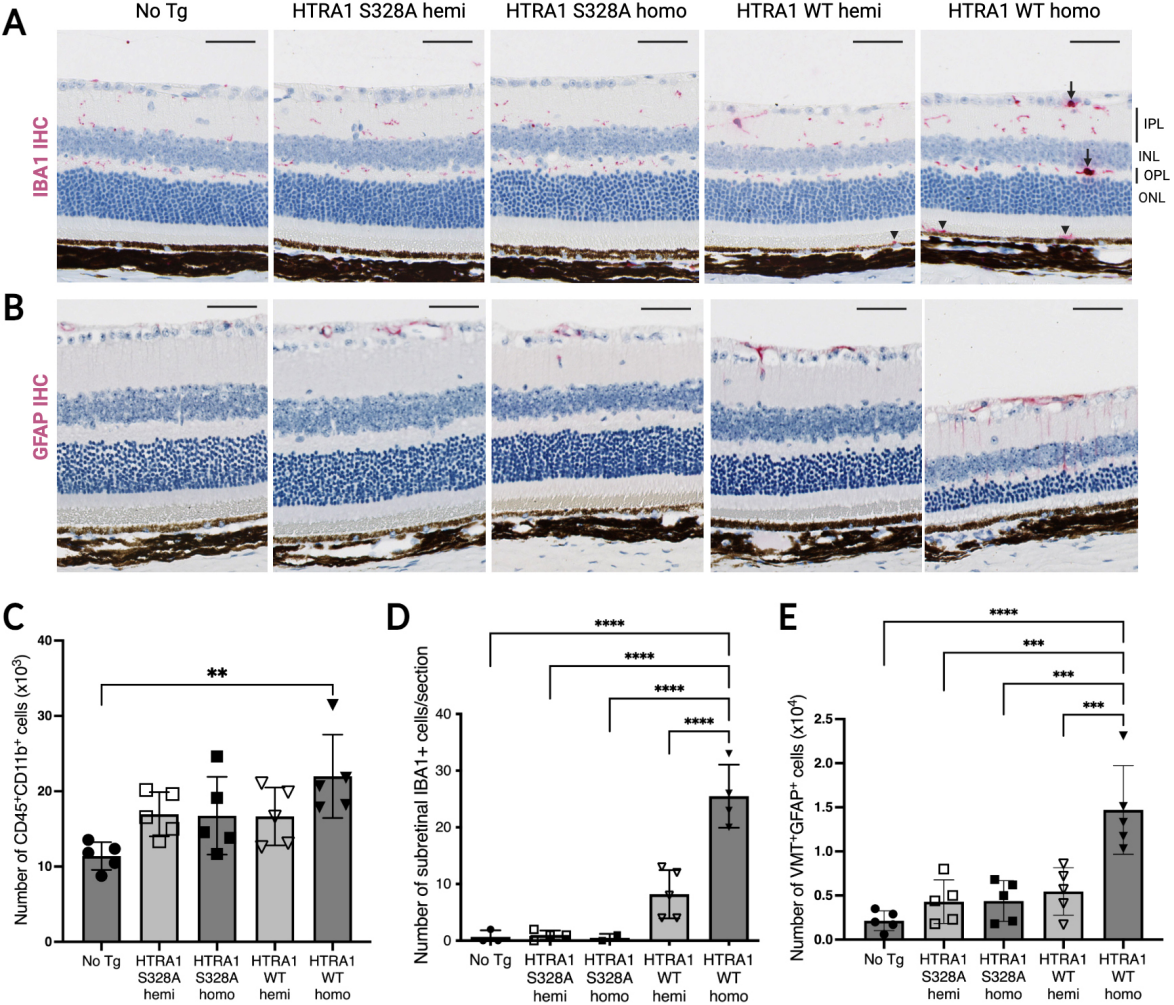
**Human HTRA1 overexpression in mice leads to reactive retinal gliosis.** (A) IHC images showing IBA1-positive cells in retinas from the different mouse strains. Mice expressing HTRA1 (WT, hemi and homo) have IBA1-positive cells in between the RPE and the photoreceptor layer (arrowheads) that are absent in the No Tg mice and mice expressing catalytic dead HTRA1 (S328A, hemi and homo). Activated microglia cells with intensified IBA-1 immunoreactivity are present in HTRA1 WT homozygous retinas (arrows). Representative images 1.5 mm away from the optic nerve head, from a cohort of 11-month-old mice, *n*=5 to 6 mice per genotype. Scale bars: 50 µm. (B) Representative IHC images showing GFAP labeling in retinas of the different mouse strains. Mice expressing HTRA1 (WT, hemi and homo) have GFAP-positive cells spanning the entire inner retina, which are absent in the No Tg mice and mice expressing catalytic dead HTRA1 (S328A, hemi and homo). Representative images 0.7 mm away from the optic nerve head, from a cohort of 11-month-old mice, *n*=5-6 per genotype. Scale bars: 50 µm. (C) Number of microglia (CD45 and CD11b double-positive cells) provided by flow cytometry analysis of the retinas of the different mouse strains. 6-month-old mice, *n*=5 mice per genotype. One-way ANOVA; **P<0.005. Representative flow cytometry data in Fig. S2. (D) Quantification of subretinal IBA1-positive cells present between the RPE and the photoreceptor outer segments in the retinas of the different mouse strains as seen in the representative images in A. 11-month-old mice, *n*=5-6 mice per genotype. One-way ANOVA; ****P<0.0001. (E) Number of activated Müller glia cells [vimentin (VMT) and GFAP double-positive cells] provided by flow cytometry analysis of the retinas of the different mouse strains. 6-month-old mice, *n*=5 mice per genotype. One-way ANOVA; ***P<0.001, ****P<0.0001. Representative flow cytometry data in Fig. S2.

Next, we examined whether the observed retinal degeneration in HTRA1 WT transgenic mice is associated with reactive gliosis of microglia and Müller glia by use of IHC and flow cytometry. Microglia in healthy retinas are usually quiescent and display a ramified morphology; they typically reside in the inner (IPL) and outer (OPL) plexiform layers and constitutively express IBA1, which is used as a pan-microglial marker. In non-transgenic mice or mice expressing catalytic inactive HTRA1 S328A, IBA1-positive cells were detected in the IPL and OPL (Fig. 3A). In HTRA1 WT homozygous mice, some of these cells showed intensified IBA1 immunoreactivity and changed to an amoeboid morphology with swollen or enlarged soma (Fig. 3A, arrows). These alterations in morphology and IBA1 protein expression are indicative of an activation state. Activated microglia also upregulate CD11b (also known as ITGAM). By flow cytometry analysis of CD11b and CD45 (also known as PTPRC), a pan-leukocyte surface marker that normally is expressed at low to moderate levels by resting microglia, we found a significant increase in activated microglia or macrophage (i.e. CD45/CD11b double-positive cells) in HTRA1 WT homozygous retinas (Fig. 3C; Fig. S2). Moreover, some IBA1-positive cells were also present between the photoreceptor outer segments and RPE in HTRA1 WT-expressing mice (hemizygous and homozygous) (Fig. 3A, arrowheads). This is characteristic of unhealthy retina and stressed photoreceptors. Quantification of these subretinally localized cells revealed a HTRA1 WT dosage-dependent increase in cell numbers, with higher numbers of subretinal IBA1-positive cells found in HTRA1 WT homozygous (25.5±5.6 cells) versus hemizygous (8.2±4.2 cells) mice (Fig. 3D). Interestingly, the subretinal IBA1-positive cell number in HTRA1 WT hemizygous and homozygous mice also increased with age, mirroring the photoreceptor degeneration progression (Fig. S1G, 6-month-old; Fig. 3D, 11-month-old).

Müller glia in normal retinas constitutively express vimentin (VMT; also known as VIM) and little or no glial fibrillary acidic protein (GFAP), which is typically restricted to astrocytes in the nerve fiber layer, as seen in non-transgenic and HTRA1 S328A-expressing mice (Fig. 3B). However, following retinal injury or stress, Müller glia undergo gliotic response and rapidly upregulate GFAP. HTRA1 WT homozygous retinas showed increased expression of GFAP, indicative of Müller glia activation (Fig. 3B). In addition, flow cytometry demonstrated that HTRA1 WT homozygous retinas had increased numbers of VMT^+^/GFAP^+^ Müller glia cells (Fig. 3E; Fig. S2).

In conclusion, reactive retinal gliosis was observed in HTRA1 WT-overexpressing mice. Subretinal microglia located below the degenerating photoreceptor layer were observed in both HTRA1 WT hemizygous and homozygous eyes. Furthermore, activated microglia cells and reactive Müller glia cells were observed in HTRA1 WT homozygous mice.

### HTRA1 overexpression does not worsen phototoxic stress but exacerbates choroidal neovascular lesions

**Fig. 4.**
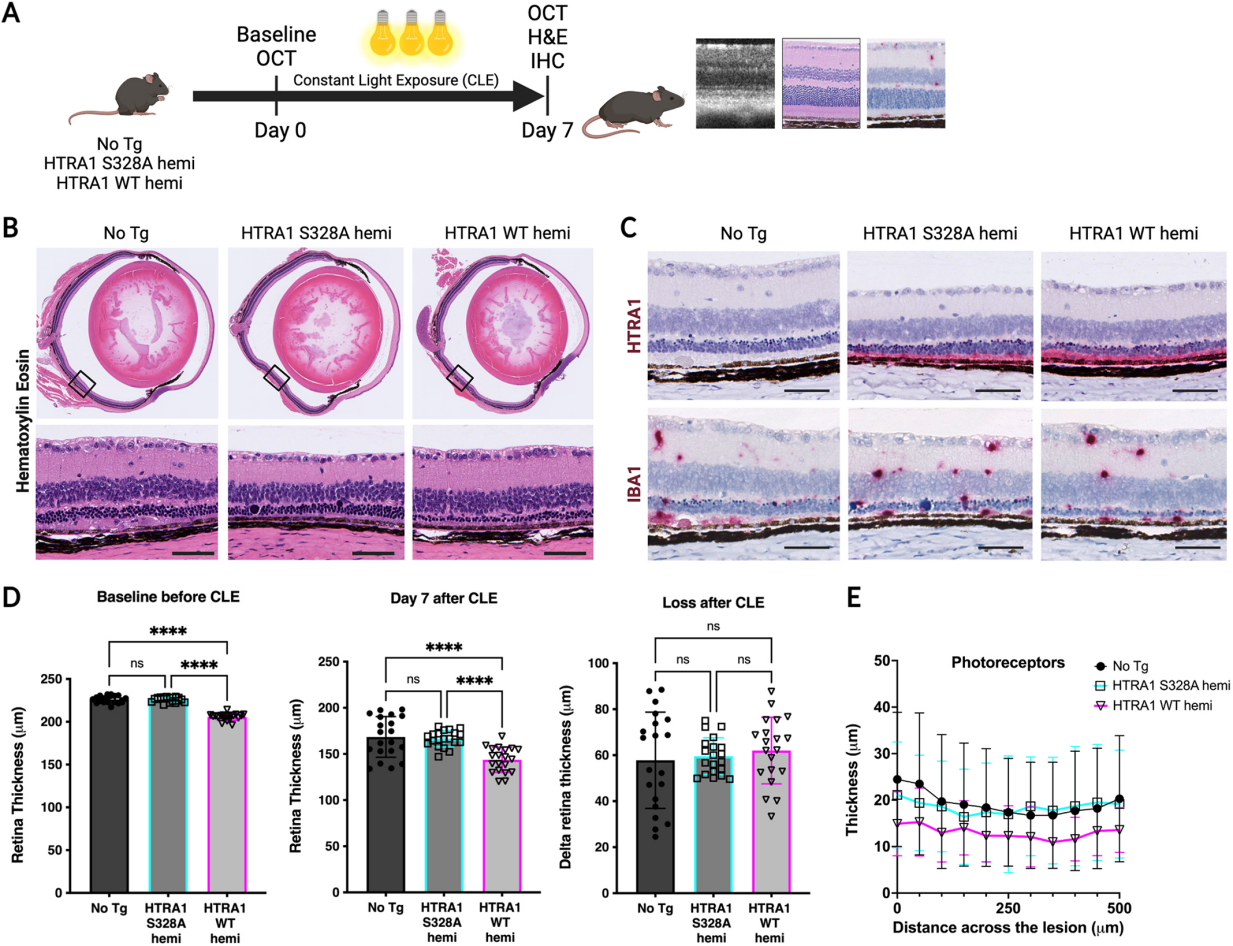
**Human HTRA1 overexpression in mice does not exacerbate phototoxic injury.** (A) Diagram of the constant light exposure (CLE) experimental design. Baseline optical coherence tomography (OCT) scans were performed on No Tg mice and mice expressing catalytic dead HTRA1 (S328A, hemi) or catalytic active HTRA1 (WT, hemi). The mice were then exposed to constant light for 1 week, after which retinas were analyzed by OCT scans, histology (H&E) or IHC. Created in BioRender by Gu, X. (2025). https://biorender.com/vgx0o71. This figure was sublicensed under CC-BY 4.0 terms. (B) Histologic characterization of retinal degeneration due to phototoxicity from CLE. Representative H&E-stained sections (top row), with insets (bottom row) showing the retinal area most affected by the CLE. Outer nuclear layer and photoreceptors are severely degenerated, similarly across genotypes. Representative images from a cohort of ten eyes per genotype. Scale bars: 50 µm. (C) IHC showing human HTRA1 protein in the retinas of HTRA1 transgenic mice (S328A and WT), but absent in the No Tg mice (top row), and IBA1 protein in the retinas of the different mouse strains (bottom row). Representative images from a cohort of ten eyes per genotype. Scale bars: 50 µm. (D) Quantification of retina thickness by OCT in the different mouse strains at baseline before the CLE (left) and after 1 week of light exposure (Day 7, center). Loss of retina thickness is expressed as the delta between the thickness at baseline and after the CLE (right). *n*=20 eyes per genotype. One-way ANOVA; n.s., not significant (*P*>0.05); ****P<0.0001. (E) Quantification of photoreceptor thickness measured across 500 µm of the CLE-induced lesions from H&E-stained sections after 1 week of light exposure. *n*=10 eyes per genotype.

Next, we sought to determine whether HTRA1 overexpression increases retinal susceptibility to AMD-related environmental stress. First, we subjected the mice to constant light exposure (CLE) for a week. This model applies bright light to cause phototoxic stress to the retina, and results in progressive loss of photoreceptors and increased activation of Müller cells and microglia (Natoli et al., 2016). On the basis that HTRA1 WT homozygous mice spontaneously develop severe loss of photoreceptors, we chose to enroll hemizygous mice to determine whether the phenotypes could be exacerbated (Fig. 4A). After 1 week of phototoxic stress, retinal thinning determined by histology was observed in all examined genotypes, i.e. the non-transgenic, HTRA1 S328A hemizygous and HTRA1 WT hemizygous mice (Fig. 4B). All mice had variable mild to severe effects caused by CLE, predominantly thinning of the outer nuclear and photoreceptor layers with degenerative changes including pyknosis and large basophilic structures, which may represent swollen nuclei. HTRA1 protein was absent from non-transgenic retinas, but accumulated in the subretinal space associated with pathology in HTRA1 WT and S328A hemizygous mice (Fig. 4C, top row). In addition, amoeboid-shaped microglia with intense IBA1 expression were found in all retinal layers but predominantly in the subretinal space and associated with pathology. However, no obvious difference was observed between different genotypes (Fig. 4C, bottom row). Retinal thickness was measured by OCT at baseline and after CLE (Day 7) across the superior and inferior retina. CLE-induced retina loss was determined per mouse as the post-CLE thickness minus the baseline retina thickness, and revealed no statistical difference between genotypes. Non-transgenic mice lost, on average, 58±21 µm (25.6% of their baseline retina), HTRA1 S328A homozygous mice lost 60±8 µm (26.4% of their baseline retina), and HTRA1 WT hemizygous mice lost 62±15 µm (30.2% of their baseline retina) (Fig. 4D). Because the CLE-induced retina loss was inconsistent across the retina and more severe in a region of the inferior retina owing to the orientation of the light source, we measured photoreceptor thickness in this lesion area on histology sections. After 7 days of light exposure, the photoreceptor thicknesses for the different genotypes were as follows: 19.3±12.8 µm for non-transgenic mice, 18.5±10.7 µm for HTRA1 S328A hemizygous mice and 13.0±5.5 µm for HTRA1 WT hemizygous mice (Fig. 4E). These findings suggest that overexpression of HTRA1 WT did not exacerbate CLE-induced retinal degeneration.

**Fig. 5.**
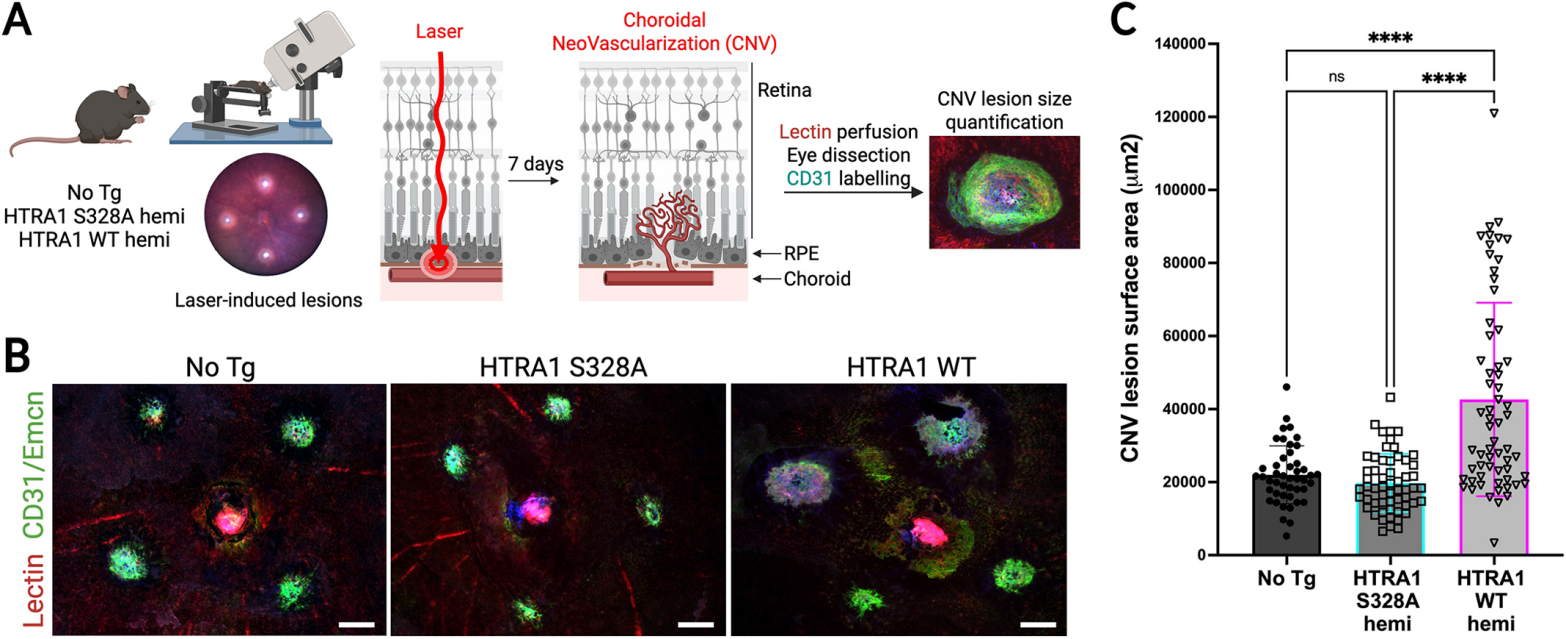
**Human HTRA1 overexpression in mice exacerbates laser-induced choroidal neovascular lesions.** (A) Diagram of the laser-induced choroidal neovascularization (CNV) preclinical model. Four laser burns per eye were performed in No Tg mice and mice expressing catalytic dead HTRA1 (S328A, hemi) or catalytic active HTRA1 (WT, hemi). CNV lesions were allowed to grow for 1 week, then lesion surface area was quantified using vascular labelling (lectin perfusion and anti-CD31 immunofluorescence). Created in BioRender by Gu, X. (2025). https://biorender.com/muizvw7. This figure was sublicensed under CC-BY 4.0 terms. (B) Representative immunofluorescence images showing lectin (red) and anti-CD31+anti-Emcn (green) labelling of four CNV lesions in an eye from a No Tg, a HTRA1 S328A or a HTRA1 WT-expressing mouse. Scale bars: 200 µm. (C) CNV lesion surface area quantification. *N*=8 to 9 mice per genotype (total of 46 to 62 lesions). One-way ANOVA; n.s., not significant (*P*>0.05); ****P<0.0001.

Next, we challenged the retinas by use of a laser-induced CNV model to mimic angiogenesis in MNV patients. In the CNV model, Bruch's membrane underneath the RPE is focally disrupted by a laser burn, thus allowing choroidal blood vessels to grow into the retina (Grossniklaus et al., 2010) (Fig. 5A). In each eye, four lesions were induced, and 1 week later the surface area of each CNV lesion was quantified using blood vessels labeling on flat mounts (Fig. 5B). HTRA1 WT hemizygous mice, but not the catalytically inactive HTRA1 S328A hemizygous mice, showed a significant increase in CNV lesion size, with mean lesion surface areas of 42,634±26,472 µm^2^, 21,981±7933 µm^2^ and 19,775±7970 µm^2^ for the HTRA1 WT hemizygous, HTRA1 S328A hemizygous and non-transgenic mice, respectively (Fig. 5C).

In conclusion, HTRA1 WT overexpression did not significantly exacerbate retinal degeneration triggered by phototoxic stress. However, it exacerbated the size of the choroidal neovascular lesions, suggesting that HTRA1 protease activity can facilitate or contribute to vascular growth in the retina.

### Identification of RBP3 as a HTRA1 substrate in the retina of HTRA1 transgenic mice

**Fig. 6.**
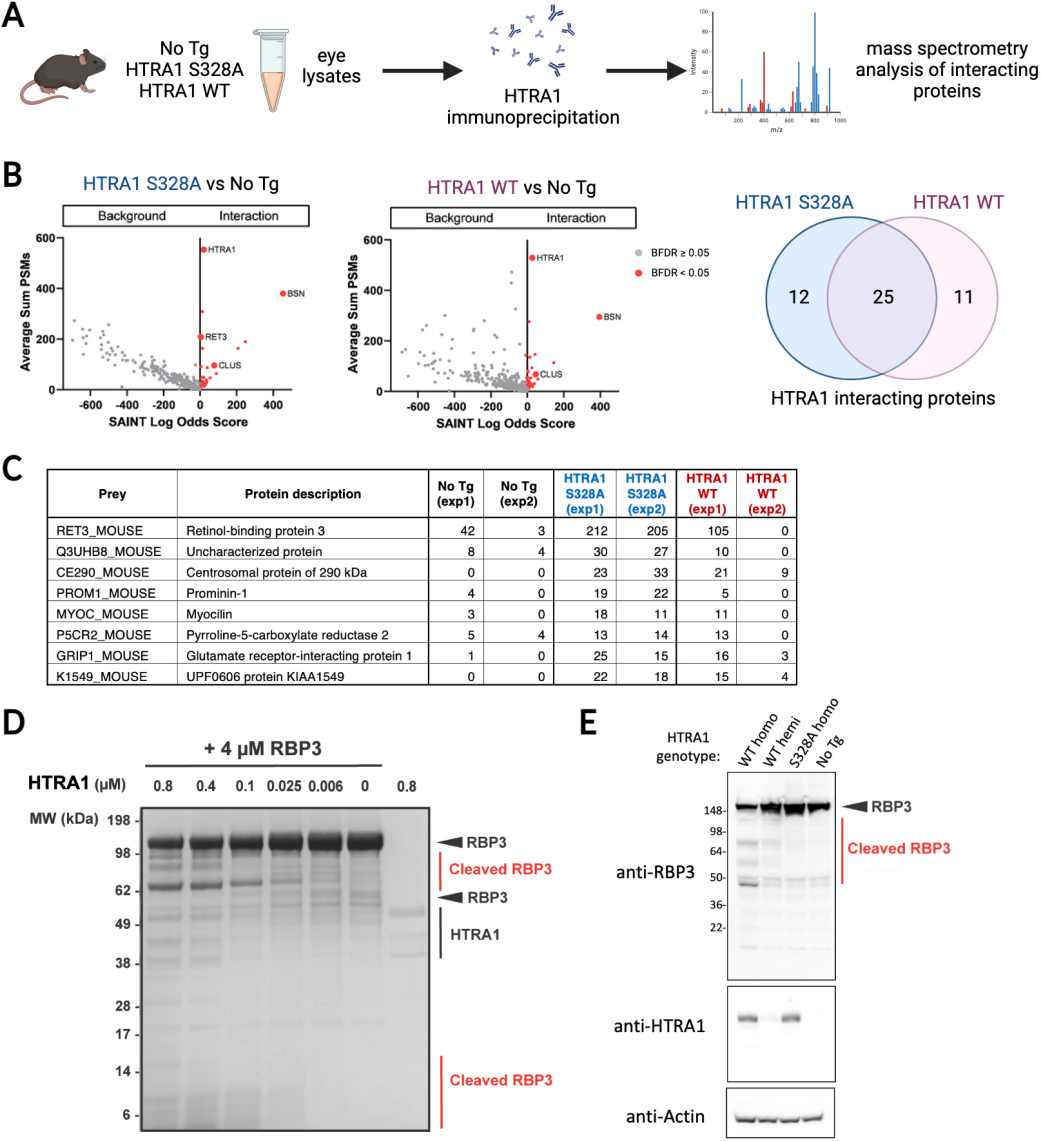
**Identification of RBP3 as a HTRA1 substrate.** (A) Diagram of the experimental design. Eye lysates from No Tg mice or mice transgenic for catalytically inactive HTRA1 (S328A) or catalytically active HTRA1 (WT) were used for HTRA1 co-immunoprecipitation. Mass spectrometry analysis was performed to identify HTRA1-interacting proteins. Created in BioRender by Gu, X. (2025). https://biorender.com/s25zoj5. This figure was sublicensed under CC-BY 4.0 terms. (B) Scatter plot representing the abundance (Average Sum PSMs) versus specificity (SAINT Log Odds Score) for all identified proteins from two independent experiments showing interacting proteins with HTRA1 S328A or HTRA1 WT. Interactions with Bayesian false discovery rate (BFDR)<0.05 were classified as significant (red), otherwise were considered as background (gray). (C) Table of the potential HTRA1 substrates defined as proteins that were more significantly enriched with HTRA1 S328A than HTRA1 WT (common contaminant keratin and proteins with low peptide counts in HTRA1 S328A were removed). The abundance of the proteins is reflected by the total peptide counts. (D) Gel stained by SimplyBlue SafeStain showing the *in vitro* RBP3 digestion assay after 24 h incubation at 37°C with different concentrations of recombinant HTRA1. The gel shown is representative of three independent experiments. MW, molecular mass. (E) Western blot of retina lysates from No Tg mice or mice transgenic for inactive HTRA1 (S328A, homo) or active HTRA1 (WT, hemi or homo) incubated with recombinant RPB3.

Our *in vivo* data presented in this study strongly support a role for HTRA1 catalytic activity in AMD-like pathologies in the retina, implying that cleavage of specific macromolecular substrates is a critical step in the observed HTRA1-mediated pathologies. Therefore, we set out to identify HTRA1 substrates by use of inactive HTRA1 S328A in an *in-vivo* ‘substrate-trapping’ approach. We used a monoclonal anti-HTRA1 antibody to immunoprecipitate HTRA1:substrate complexes from eye lysates (including retinas and RPE/choroids) of mice expressing the catalytically inactive HTRA1 S328A in comparison to lysates from non-transgenic and active HTRA1 (WT)-expressing mice. We then employed semi-quantitative mass spectrometry analysis to assess the differential interacting partners of HTRA1 S328A and HTRA1 WT (Fig. 6A). Any proteins that maintained the interactions with the inactive S328A were considered as potential substrates of HTRA1. We performed the immunoprecipitation from mouse retinal lysates in two bioreplicates in order to assess reproducibility and identified a total of 2313 proteins (Table S1). Next, we performed SAINT analysis using SAINTexpress (Teo et al., 2014) to identify significant interactions. SAINT analysis of the comparison between interacting partners of HTRA1 S328A and non-transgenic mice identified 37 significant interacting proteins for HTRA1 S328A (Table S2), while the comparison between HTRA1 WT and non-transgenic mice resulted in 36 significant interacting proteins for HTRA1 WT (Table S3). Among the high-abundance proteins that were identified as interacting partners of both HTRA1 WT and HTRA1 S328A were protein bassoon (BSN), clusterin (CLUS; also known as CLU), C-terminal-binding protein 1 (CTBP1) and C-terminal-binding protein 2 (CTBP2) (Fig. 6B). Upon close examination of the interacting partners of HTRA1 WT and HTRA1 S328A, 12 proteins were found to be more enriched in HTRA1 S328A samples. Excluding keratins, the common contaminants and proteins with low peptide counts resulted in eight proteins that were more enriched in HTRA1 S328A samples. Notably retinol-binding protein 3 (RBP3; also known as RET3 and IRBP; OMIM #180290) was consistently more enriched in HTRA1 S328A in both replicates compared to the HTRA1 WT samples (Fig. 6C). RBP3 is a lipophilic glycoprotein essential for vertebrates' vision. It is secreted by photoreceptors and recycled by the RPE as it facilitates transfer of retinoids in the visual cycle (Gonzalez-Fernandez, 2003; Wu et al., 2007). RBP3 also helps with lipid transport between the RPE and photoreceptors, and with maintaining retinal redox balance (Zeng et al., 2020). We first validated RBP3 cleavage by HTRA1 in an *in vitro* assay using recombinant proteins. We found that RBP3 is degraded and smaller cleavage products appear when incubated with HTRA1 (Fig. 6D). Then, we validated whether RPB3 is cleaved by HTRA1 present in the retina of HTRA1 WT transgenic mice using an *ex vivo* assay. Retinas from non-transgenic, HTRA1 S328A homozygous and HTRA1 WT hemizygous and homozygous mice were dissected, homogenized and incubated with a HTRA1-specific activity-based probe (Tom et al., 2020). As expected, HTRA1 protease activity was only detected in retinas isolated from HTRA1 WT mice, and activity was higher in homozygous mice than in hemizygous mice (Fig. S1H). Upon incubation of retina lysates with recombinant RBP3, we observed cleavage of RBP3 in the samples from HTRA1 WT mice (hemizygous and homozygous) and not in the samples from non-transgenic and HTRA1 S328A mice (Fig. 6E), validating RBP3 as a HTRA1 substrate.

In conclusion, by implementing a mass spectrometry-based substrate trapping strategy, we were able to identify several potential HTRA1 substrates in the mouse retina, including the visual cycle protein RBP3, which was further validated by using *in vitro* and retinal *ex vivo* assays.

## DISCUSSION

In this study, we confirmed that HTRA1 protein is predominantly present in blood vessels, Bruch's membrane and near horizontal cells at the IPL/ONL interface of healthy human retinas. Using donor eyes from patients with different AMD stages, we showed that HTRA1 protein starts to accumulate in the extracellular matrix surrounding the RPE at the early stage of the disease (iAMD stage). As the disease progresses, the accumulation intensifies, and HTRA1 protein build-up is associated with degenerating RPE cells (GA stage), before disappearing in the fibrous scar present at the final stage of the disease (end-stage GA). It would be interesting to identify the origin of accumulated HTRA1 proteins and to determine whether they are coming locally from RPE cells or are secreted from distant retinal cells and accumulate at lesion sites.

Although the significance of HTRA1 production by horizontal cells is unknown, because the level of HTRA1 accumulation at the IPL/ONL interface was not altered in disease, we focused on the role of HTRA1 around the RPE, for which we saw changes with disease progression. We developed a transgenic mouse model in which human HTRA1 is expressed and secreted by RPE, recapitulating HTRA1 protein accumulation in Bruch's membrane in human eyes. To specifically assess the role of HTRA1 protease activity, we generated the corresponding transgenic mice expressing a catalytically inactive form of human HTRA1 that retains other potential functions of the protein, such as a putative role as a chaperone [HTRA1 S328A (Hu et al., 1998)]. We used these transgenic mouse strains to determine whether overexpression of HTRA1 increases susceptibility to factors known to contribute to AMD, such as aging, environmental stress (i.e. excessive exposure to light) or Bruch's membrane instability. We found that mice expressing protease-active HTRA1 spontaneously developed age-related photoreceptor degeneration and loss of function. This phenotype was specifically dependent on HTRA1 protease activity, as it was absent in the protease-inactive HTRA1 S328A mice. It was also dependent on the level of HTRA1 protein, as it was more severe in HTRA1 WT homozygous mice than in hemizygous mice. Because mice overexpressing HTRA1 had a spontaneous phenotype, HTRA1 accumulation in the eyes of patients with AMD could contribute to pathology progression without the requirement for specific additional pathogenic factors increasing HTRA1 activity. This aligns with the fact that HTRA1 protease activity can be regulated by allosteric activation following substrate binding (Cabrera et al., 2017; Truebestein et al., 2011).

It has been described that HTRA1 overexpression can be deleterious for RPE functions, such as phagocytosis, and this can indirectly affect photoreceptor survival (Melo et al., 2018). However, the herein observed accumulation of overexpressed HTRA1 in the interphotoreceptor matrix could directly perturb photoreceptor function and survival, for instance by HTRA1-mediated degradation of the important visual cycle protein RBP3, which was identified in retinal lysates by use of a substrate trapping strategy. We previously identified RBP3 as one of the potential HTRA1 substrates in the vitreous of rabbit and cynomolgus monkey by using an orthogonal proteomics approach, i.e. the terminal amine isotopic labeling of substrates (Tom et al., 2020), further corroborating RBP3 as a physiologically relevant HTRA1 substrate.

RBP3 downregulation has been observed in many animal studies and occurs in the early stages of various retinal diseases (Malechka et al., 2017; Narfström et al., 1989; Zeng et al., 2020; Zheng et al., 2015; Zhu et al., 2015). RBP3 mutation, dysfunction or downregulation were also described in several human eye diseases in which photoreceptors are affected (Arno et al., 2015; Georgiou et al., 2024; den Hollander et al., 2009). Lower levels of functional RBP3 can disrupt the visual cycle, which can then lead to accumulation of toxic lipofuscin. Thus, the role of RBP3 in preventing lipofuscin build-up could be central for AMD-like diseases (Radu et al., 2003). Interestingly, RBP3 upregulation has been shown to prevent photoreceptor degeneration in diabetic rodents through VEGF inhibition (Yokomizo et al., 2019), suggesting that RBP3 could also be beneficial in diseases characterized by photoreceptor loss and VEGF dysregulation, such as AMD. It would be interesting to probe whether, in patients with AMD, increased HTRA1 at the lesion site correlates with a decrease in RPB3, due to excessive enzymatic digestion.

Using our transgenic mice, we showed that overexpression of protease active HTRA1 triggered spontaneous age-related photoreceptor degeneration; however, this retinal degeneration was not exacerbated when the mice were subjected to phototoxic stress under the CLE preclinical model. One explanation could be that overexpression of HTRA1 affects the same biological pathway that is affected by CLE, resulting in the absence of cumulative effect. Interestingly, RPB3 is critical for the visual cycle, and it has been reported that *Rbp3* mRNA levels are reduced in the light-induced retinal degeneration rat model (Wong et al., 2001). If lower RBP3 levels are associated with photoreceptor degeneration in the CLE model, it is possible that, in HTRA1-overexpressing mice, RBP3 levels are already lower and there is therefore no exacerbation. In contrast, mice overexpressing protease-active HTRA1 did not develop spontaneous CNV but showed significant worsening of laser-induced CNV lesions, suggesting that HTRA1 protease activity promotes, rather than initiates, vascular growth in the retina. However, it has been reported that another transgenic mouse line overexpressing human HTRA1 in the RPE spontaneously develops PCV and destabilization of Bruch's membrane with elastin degradation (Jones et al., 2011; Kumar et al., 2014; Navneet et al., 2024). It is interesting to note that our mouse transgenic line was on a different genetic background (C57BL/6 instead of CD1) and elastin was not identified in our HTRA1 substrate screen, potentially explaining the absence of spontaneous PCV in our mouse line.

In conclusion, our data showed that increased HTRA1 at the RPE/photoreceptor interphase is deleterious for the retina, causing AMD-like phenotypes such as age-related photoreceptor degeneration and exacerbation of choroidal neovascularization. This clarifies that overexpressing HTRA1 in the retina is not a viable therapeutic approach and, instead, supports the therapeutic hypothesis that HTRA1 protease inhibition could be beneficial for patients with AMD.

## MATERIALS AND METHODS

### Ethics statement

The studies involving humans were approved by Lions Eye Institute for Transplant and Research ethics committee and conducted in accordance with the local legislation and institutional requirements. All eyes were obtained after written consent and used in accordance with the guidelines of the Declaration of Helsinki for research involving the use of human tissue. The animal studies were approved by Genentech Institutional Animal Care and Use Committee (IACUC) and conducted in accordance with the local legislation and institutional requirements.

#### Human donor eyes

Postmortem human donor eyes were obtained from the Lions Eye Institute for Transplant and Research in Tampa, FL, USA. Whole globes were fixed in 10% neutral-buffered formalin for 24 h, then transferred into 70% ethanol until paraffin embedding. Eyes were sectioned transversely at 4 µm thickness throughout the entire macula, and every 30th section was stained with Hematoxylin and Eosin (H&E) to confirm the diagnosis of either unaffected control, early/intermediate AMD or GA. Slides adjacent to those showing disease-defining features were used for IHC using the Dako autostainer platform. Ten eyes from six unaffected donors (four males and two females) of age range of 51-89 years, and 11 eyes from six patients with AMD (one male and five females) of age range of 50-90 years, were used for the IHC analysis. Formalin-fixed paraffin-embedded sections were deparaffinized and heat antigen retrieved with Dako Target Retrieval (Agilent, S1700) for 20 min at 99°C. The sections were blocked with 3% bovine serum albumin, followed by 5 µg/ml mouse anti-human HTRA1 (Genentech, clone 4B1) or rabbit anti-human CD31 (Bethyl Labs, polyclonal) for 60 min. Signal was detected with Powervision polymer-AP anti-Ms (Leica Biosystems, PV6110) for 30 min and visualized with Fast Red (ScyTek, FR0001-1FU). Slides were counterstained with Mayer's Hematoxylin (Rowley Biochemical, L-756-1A) for 1 min followed by bluing reagent.

The anti-HTRA1 antibody clone 4B1 was developed as previously described (Tom et al., 2020). Briefly, mice with knockout of *Htra1* were immunized with recombinant muHTRA1, then isolated mouse spleen cells were fused with myeloma cells for hybridoma screening by enzyme-linked immunosorbent assay (ELISA) to identify clones cross-reacting with both mouse and human HTRA1.

#### Mouse transgenic design and genotyping

In the mouse transgenic HTRA1 WT, the construct consists of a human *VMD2* promoter (Esumi et al., 2004) followed by 75 bp small-mcs sequence from pGL2 and 1.4 kb of human *HTRA1* cDNA until the stop codon (TAG), followed by BGH pA. In the mouse transgenic HTRA1 S328A, the same construct was inserted with the exception of a missense point mutation in the *HTRA1* gene (TCG to GCG) to create the S328A substitution in exon 2. The transgene plasmid vector (5 µg) was linearized overnight to remove the vector backbone and loaded onto a 0.8% 1× Tris-borate-EDTA (TBE) agarose gel. The band of interest was excised from the gel, and Zymoclean Gel DNA recovery kit was used to extract the DNA. Approximately 500 ng of recovered DNA was run again on a 0.8% 1× TBE agarose gel to check for complete digestion and purity. Transgenic DNA was diluted to 1 ng/µl in the microinjection buffer before proceeding to microinjection into C57BL/6J zygotes. Founder mice were bred to C57BL/6J, and tail DNA from offspring was used for digital droplet PCR (ddPCR) to assess the copy number of the transgene. A TaqMan assay was used to screen for the presence or absence of the *HTRA1* transgene in the production colony using the following primers: VMD2-HTRA1 F, 5′-CCACTTACGAAGCCAAAA-3′; VMD2-HTRA1 R, 5′-CGACCACGAACTCTCC-3′; VMD2-HTRA1 Pr/FAM, 5′-AGCAGACATCGCACTCATC-3′.

#### Animal maintenance, examination and ocular preclinical models

All animal experiments were approved by the Genentech IACUC and conducted in compliance with the Institute for Lab Animals' guidelines for the humane care and use of laboratory animals, and were adherent to the Association for Research in Vision and Ophthalmology Statement for the Use of Animals in Ophthalmic and Vision Research. Animals were housed with *ad libitum* access to food and water and subjected to a 14 h light/10 h dark cycle, except for the animals subjected to the CLE model. Both males and females were used for experiments, and littermates were used as controls in all experiments.

For all the ocular examination procedures, mice were anesthetized by intraperitoneal injection of ketamine (70-80 mg/kg body weight) and xylazine (15 mg/kg body weight). Pupils were dilated with drops of Tropicamide Ophthalmic Solution USP 1% (Akorn), and drops of Systane lubricant eye drop (Alcon) were used to prevent corneal dehydration. After ocular examination, anesthetized mice were placed on a pre-warmed plate at 37°C until they were ambulatory.

Ocular fundus images were obtained using a Spectralis HRA+OCT system (Heidelberg Engineering) modified according to the manufacturer's recommendations with a 55° widefield lens placed in front of the camera to adjust for rodent optics.

Fluorescein angiography was also performed with the Spectralis HRA+OCT system (Heidelberg Engineering). After anesthesia, mice were intraperitoneally injected with fluorescein AK-FLUOR (Akorn) at 5 μg/g body weight in physiological saline. Then, 5 and 10 min after the injection, images were acquired with a 488 nm light filter.

Retinal thickness was measured by OCT scanning using a Bioptigen Envisu R machine (Leica Microsystems). Total retina thickness was automatically determined using an algorithm (Matlab software, MathWorks) and defined as the width from the nerve fiber layer to the RPE/choroid layer on the cross-sectional scans (whole mouse retina scans 1.8×1.8 mm, with 1.6 µm axial resolution and an exclusion of 100 µm at the optic nerve head).

ERG recordings were performed using the Celeris electrophysiology system (Diagnosys) after overnight dark adaptation of the mice, and all procedures were performed under low-level red light. A reference electrode was inserted subcutaneously through the forehead, and a ground electrode was inserted subcutaneously at the base of the tail. Electrodes with a light stimulator were placed on both eyes. Under scotopic conditions, eyes were stimulated with six flash intensities (0.001, 0.01, 0.05, 0.1, 2 and 4 cd.s/m^2^). After 5 min of light adaptation, eyes were then stimulated with photopic flash of four intensities (2, 5, 50 and 250 cd.s/m^2^). Recorded signals were bandpass filtered at 0.15-1000 Hz and sampled at 2 kHz. All of the recorded data points were analyzed using a custom Matlab software (MathWorks). Responses to three to five flashes of light stimulation were averaged.

For the CLE retinal degeneration model, animals were administered with pupil-dilator eye drops twice daily (1% Atropine Sulphate, Akorn) and were housed in transparent plastic boxes and exposed to 100,000 lux of white LED lighting (measured using an Extech HD450 light meter) for 24 h per day for 7 days. Cages were rotated daily within each shelf and between shelves to ensure equal light exposure.

For the laser-induced CNV injury model, animals received analgesic (buprenorphine, 0.05 mg/kg) intraperitoneally and were then anesthetized by intraperitoneal injection of ketamine (70-80 mg/kg body weight) and xylazine (15 mg/kg body weight). Pupils were dilated with drops of 1% Tropicamide Ophthalmic Solution USP (Akorn). Neovascularization was induced in each eye using an image-guided laser system (Micron III, Phoenix Research Laboratories) with a 532 nm wavelength laser (spot size, 100 μm; power, 320 mW; duration, 80 ms). Four burns per eye were made ∼200-300 μm away from the optic nerve. Cases of hemorrhage induced by the laser were excluded from the analysis. After the procedure, a topical antibiotic (Neomycin and Polymyxin B Sulfates and Bacitracin Zinc Ophthalmic Ointment, Bausch & Lomb) was applied to both eyes, and the mice were placed on a pre-warmed warming plate at 37°C until they were fully awakened. Seven days after the laser induction, Dylight 594-conjugated Tomato Lectin (0.1 ml of 1 mg/ml, Vector Laboratories) was intravenously injected and circulated for 5 min before eyes were collected and fixed in 4% paraformaldehyde (PFA) for 2-3 h. Eyecup/sclera flatmounts were prepared, stained for CD31 (BD Biosciences, 550274; 1:200) and endomucin (Emcn) (Invitrogen, 14-5851-82; 1:200), followed by donkey anti-rat Alexa Fluor 488 (Invitrogen, A21208; 1:500) and mounted in DAPI Fluoromount-G (SouthernBiotech; 0100-200). The CNV lesions were outlined on fluorescent microscopy images, and the surface area of each lesion was quantified using the Imaris or ImageJ software.

#### Histology, IHC and immunofluorescence analysis of mouse eyes

Mouse eyes were fixed in Davidson's fixative (Electron Microscopy Sciences) or 4% PFA for 24 h, immersed in 70% ethanol and then processed for paraffin embedding and sectioning. H&E staining was performed on sections from the eyes fixed with Davidson's fixative according to standard protocol. IHC staining of sections from the eyes fixed with PFA was performed on a Dako Autostainer platform. After rehydration, sections were treated in Dako Target Retrieval Solution. Sections were incubated with a mouse monoclonal anti-human HTRA1 antibody (Genentech, clone 4B1; 2.5 μg/ml), a rabbit polyclonal anti-GFAP antibody (Dako, Z0334; 1:500), a rabbit polyclonal anti-IBA1 antibody (Wako Chemicals, 019-19741; 0.5 μg/ml) or negative control antibodies in blocking buffer for 1 h. After washing, the sections were incubated with PowerVision Polymer-AP anti-mouse IgG and Polymer-AP anti-rabbit IgG (Leica Biosystems) for 30 min, followed by detection with Fast Red/Naphthol Phosphate reagent (ScyTek). After being counterstained with Hematoxylin, the sections were imaged with brightfield microscopy. Myeloid cell quantification was performed by manually counting the IBA1-positive cells in each retinal layer along the full length of retinal sections cut in the vertical meridian, including the optic disc.

For morphometric analysis of the retina layer thickness, 4 μm sections of mouse eyes covering the entire retina, including the optic nerve, were stained with H&E. Slides were scanned using an Olympus Nanozoomer 2.0 HT digital slide scanner (Hamamatsu) running NDP Scan software with an Olympus Uplan SApo 0.75 NA 20× objective lens, and images were analyzed using Matlab (MathWorks). Retina thickness was measured every 250 μm, up to 2000 μm on each side of the optic nerve head.

For branching point quantification of retinal blood vessels, 58- to 72-week-old mouse eyes were fixed in 4% PFA for 2 h then rinsed in PBS. Retinas were dissected intact from the globe, incubated in the blocking buffer for 4 h then incubated with an anti-COL4 antibody (Thermo Fisher Scientific, PA1-28117; 1:400) for 48 h. After three washes, the retinas were incubated with a secondary antibody, Alexa Fluor 488 (Invitrogen; 1:500), for 24 h. After three washes, the retinas were flat mounted using Mowiol containing DAPI for nucleus staining. Branching points on the primary arteries and veins of the retinas were quantified using a fluorescent microscope.

#### Processing of mouse eyes for ELISA, western blotting and flow cytometry

After dissection of the anterior chamber and lens removal, mouse retinas and RPE/choroids were isolated, rinsed in PBS, and either dissociated for fluorescence-activated cell sorting (FACS) analysis or homogenized in the cell lysis buffer (Cell Signaling Technology) using a tissue homogenizer (IKA) for ELISA and western blotting.

The retina and RPE/choroid homogenate was centrifuged at 14,000 ***g*** for 10 min at 4°C, and the supernatant was collected. HTRA1 protein concentrations in the retina lysates and RPE/choroid lysates were measured using a human HTRA1 ELISA kit (R&D Systems) 1C9/MAB2916-bio. HTRA1 concentrations were normalized to total protein content measured by BCA assay (Thermo Fisher Scientific).

For flow cytometry, retinas were isolated and digested with Earle's balanced salt solution (EBSS) containing 20 IU/ml papain and 200 IU/ml DNase (Worthington Biochemicals) for 30 min at 37°C. After gentle pipetting to dissociate the cells, papain digestion was terminated by resuspension in EBSS containing ovomucoid protease inhibitor (Worthington Biochemicals). Total retinal cells were quantified using a LSRFortessa flow cytometer (BD Biosciences). Live cells were gated on propidium iodide-negative cells. Cells were resuspended in flow cytometry buffer (PBS containing 0.5% bovine serum albumin and 2 mM EDTA, pH 8.0) and incubated with anti-CD16/CD32 (BD Biosciences) for 30 min to block any nonspecific binding to Fc receptors. Retinal cells were then stained with PE-Cy7-conjugated anti-CD11b (BD Biosciences, clone M1/70), APC-conjugated anti-CD90.2 (BD Biosciences, clone 53-2.1), Alexa Fluor 700-conjugated anti-CD45 (BioLegend, clone 30-F11), FITC-conjugated anti-ST2 (MD Bioproducts, clone DJ8) and PE-conjugated anti-CCR2 (R&D Systems). To detect intracellular markers, the following fluorophore-conjugated antibodies were generated using antibody conjugation kits (Abcam) according to the manufacturer's instructions: PE-Cy7-conjugated anti-rhodopsin (Rho; EMD Millipore, clone 1D4), PE-conjugated anti-cone arrestin (CAR; EMD Millipore) and PerCP-Cy5.5-conjugated anti-GFAP (Thermo Fisher Scientific, clone GA5). Alexa Fluor 647-conjugated anti-vimentin (clone D21H3) was purchased from Cell Signaling Technology. Cells were stained with violet fixable viability dye (Life Technologies), fixed, and permeabilized with IntraPrep permeabilization reagent (Beckman Coulter) according to the manufacturer's instructions. Cells were then stained with the antibody cocktail for 30 min, washed and analyzed on the LSRFortessa flow cytometer. All data were acquired with BD FACSDiva software and analyzed with FlowJo software (GraphPad). Total numbers of rods (Rho^+^CAR^−^), cones (Rho^−^CAR^+^), ganglion cells (CD90^+^CD45^−^), microglia (CD45^low^CD11b^+^) and macrophages (CD45^high^CD11b^+^) were calculated by multiplying the percentage of each cell type with total live cells.

For western blotting, retina lysates were separated by electrophoresis on Novex SDS-Tris-Glycine polyacrylamide gels (Life Technologies) and transferred to nitrocellulose membranes using the iBlot system (Invitrogen). After blocking, the membranes were probed with anti-RBP3 (Proteintech, 14352-1-AP), anti-actin (Thermo Fisher Scientific, MA5-15739; 1:2000) and anti-HTRA1 (NovusBio, 701-000; 1:3000), followed by probing with appropriate HRP-conjugated secondary antibodies (Jackson ImmunoResearch). Blots were processed using ECL Plus Western blot detection reagents (GE Healthcare). For HTRA1 activity assessment, a HTRA1-specific activity-based probe (ABP) was added to the retina homogenates for 35 min at 25°C before proceeding with the western blot. The ABP used is a Leu-Val dipeptide with a phosphonate warhead at the C-terminus and an N-terminal PEG linker with a TAMRA fluorophore. This probe was described previously (Tom et al., 2020). ABP fluorescence activity was detected by Typhoon Imager.

#### In-gel reduction/alkylation and tryptic digestion

In preparation for mass spectrometry analysis, samples were separated on 4-12% Bis-Tris gel (Thermo Fisher Scientific, NW04120) under reduced conditions. Proteins were stained with SimplyBlue stain (Invitrogen) and de-stained in water. The gel was excised from top to bottom into 15 bands per lane. Gel pieces were further de-stained in 50 mM ammonium bicarbonate (NH_4_HCO_3_)/30% acetonitrile (ACN) and dehydrated in 100% ACN. In-gel tryptic digestion was performed by rehydrating the gel pieces in 10 ng/µl trypsin solution in 25 mM NH_4_HCO_3_ and chilled on ice for 1 h. Excess trypsin solution was removed, and digestion was performed overnight in 25 mM NH_4_HCO_3_ at 37°C. Peptides were extracted with 0.1% trifluoroacetic acid in ACN. The peptide digests were dried to completion in the SpeedVac and re-suspended in 2% ACN/0.1% formic acid (FA)/water.

#### Liquid chromatography–MS/MS analysis

Dried peptide digests were reconstituted in solvent A (2% ACN/0.1%FA/water) and injected via an auto-sampler for separation by reverse-phase chromatography on a NanoAcquity UPLC system (Waters, Dublin, CA, USA). Peptides were loaded onto the Symmetry^®^ C_18_ column (1.7 mm BEH-130, 0.1×100 mm, Waters) with a flow rate of 1.5 µl/min. A gradient of 2% solvent B to 25% solvent B (solvent A is 0.1% FA/2% ACN/water and solvent B is 0.1% FA/2% water/ACN) was applied over 35 min with a total analysis time of 60 min. Peptides were eluted directly into an Advance CaptiveSpray ionization source (Michrom BioResources/Bruker, Auburn, CA, USA) with a spray voltage of 1.3 kV and were analyzed using an LTQ Orbitrap Elite mass spectrometer (Thermo Fisher Scientific). Precursor ions were acquired in the Fourier-transform mass spectrometry at 60,000 resolution; tandem mass spectrometry (MS/MS) was performed in the LTQ, with the instrument operated in data-dependent mode, whereby the 15 most abundant ions were selected for fragmentation in each duty cycle.

#### Mass spectrometry data analysis and statistical analysis

For peptide identification, MS/MS spectra were searched using the search algorithm Mascot (Matrix Sciences, London, UK) against the concatenated target-decoy database comprised of UniProt mouse protein sequences (UniProt, version 2011_12), known contaminants and the reversed versions of each sequence. A 50 ppm precursor ion mass tolerance and 0.8 Da fragment ion tolerant were selected with tryptic specificity with up to three miscleavages. Variable modifications were permitted for methionine oxidation (+15.9949 Da), propioamide adduct for cysteine residues (+71.0371 Da). Peptide assignments were first filtered to a 1% false discovery rate (FDR) at the peptide level and subsequently at 2% FDR at protein level. We used SAINTexpress (Significance Analysis of INTeractome), which is a statistical method for probabilistically scoring protein-protein interaction data from affinity purification–mass spectrometry experiments (SAINTExpress-spc v.3.6.1) (Teo et al., 2014). We compared spectral counts for each bait immunoprecipitation (IP) sample (Tg-HTRA1WT or Tg-HTRA1 S328A) and control IP sample (non-transgenic) and to assign confidence scores to observed protein–protein interactions. Protein spectral counts for each sample were calculated at the sum from the peptide spectral counts (all modified and shared peptides) for one protein in a given sample, across all fractions from the gel electrophoresis followed by liquid chromatography–mass spectrometry experiment. No filtering for protein spectral counts was performed. A single peptide is also used for protein quantification. The default setting for SAINTexpress was used (low Mod, 0; minFold, 1; normalization, 1). Control or bait samples were not compressed. Interactions with a Bayesian FDR (BFDR)<0.05 were marked as significant.

#### *In vitro* RBP3 digestion assay

*In vitro* RBP3 digestion assays were performed using recombinant human RBP3 and HTRA1 proteins. cDNA encoding human HTRA1 Q23-P480 was cloned into N-terminal TEV cleavable His6 tag vector for expression in *Trichoplusia ni* cells, and recombinant HTRA1 trimer was purified as previously described (Gerhardy et al., 2022). Human *RBP3* cDNAs encoding amino acids T20-L1247 were cloned into a mammalian expression vector (pRK-sec) with C-His8 tag and expressed in Chinese hamster ovary cells. The protein was purified by nickel affinity chromatography and further purified on a Superdex S200 size exclusion column.

To set up the experiment, 4 μM human RBP3 was incubated with various concentrations of human HTRA1 protein for 24 h at 37°C in the cleavage buffer (200 mM NaCl, 50 mM Tris-HCl pH 8.0, 0.25% CHAPS). Digested products were reduced and run on Bolt 12% Bis-Tris gels (Thermo Fisher Scientific) in 1× MES SDS Running buffer (Thermo Fisher Scientific). Gels were stained using SimplyBlue SafeStain (Thermo Fisher Scientific), and images were acquired using a Bio-Rad GelDoc Imager System.

#### Statistical analysis and software

Statistical analyses were performed using GraphPad Prism 9. Means±s.d. are shown on all graphs. Exact values of numbers of samples used are described in Results or figure legends.

## Supplementary Material

10.1242/dmm.052253_sup1 Supplementary information

Table S1. HTRA1 immunoprecipitation from mouse retinal lysates identified 2313 proteins.

Table S2. SAINT analysis of the comparison between interacting partners of HTRA1 S328A and No Tg identified 37 significant interacting proteins for HTRA1 S328A.

Table S3. SAINT analysis of the comparison between interacting partners of HTRA1 WT and No Tg identified 36 significant interacting proteins for HTRA1 WT.
