# Supplementary material for: HTRA1-dependent proteolysis induces age-related retinal degeneration and exacerbates choroidal neovascularization
Source: Dis Model Mech. 2025 Sep 17;18(10):dmm052253. doi: 10.1242/dmm.052253 (PMC12486214; doi:10.1242/dmm.052253)
Supplement: Supplementary information [file dmm-18-052253-s1.pdf]

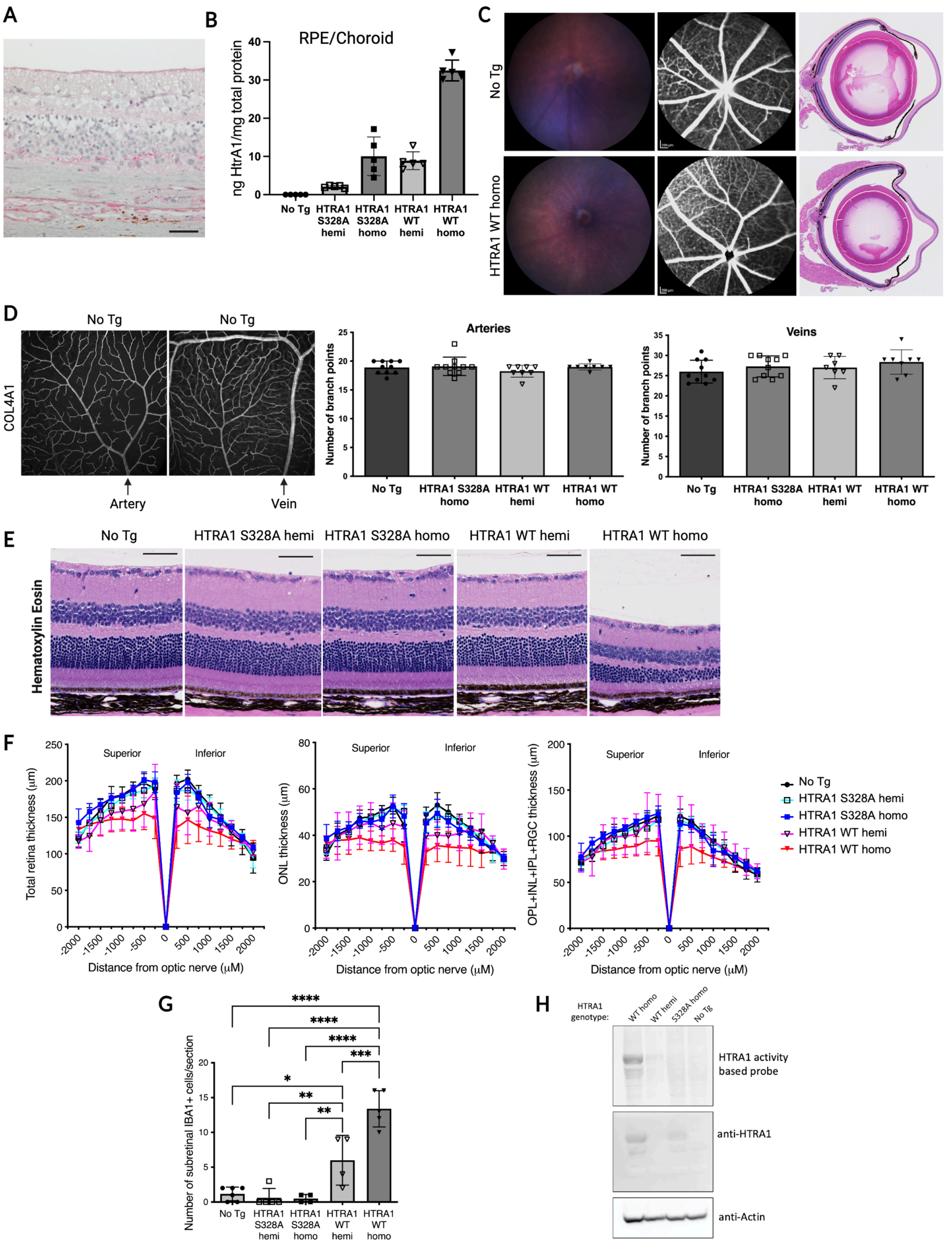

Fig. S1. Supplemental information to Figures 1 to 6.

(A) Immunohistochemistry showing that there is no appreciable HTRA1 in the fibrous scar tissue of end-stage geographic atrophy (GA). Scale bar = 50µm. **(B)** Human HTRA1 protein levels in the retina pigment epithelium

Disease Models & Mechanisms • Supplementary information

(RPE) and choroid of the different mouse strains, normalized to the total protein amounts and detected using an Enzyme-Linked Immunosorbent Assay (ELISA). n = 5 per genotype. **(C)** No gross anomalies were detected in 9-months old mice by fundus photography (left), fluorescence angiography (center) or histology, except retinal thinning, in HTRA1 WT homozygous compared to non-transgenic (No Tg) mice. **(D)** No gross anomalies were observed in retinal blood vessel patterning as assessed by branch points quantification of main arteries and veins in HTRA1 transgenic mice (inactive HTRA1 S328A and active HTRA1 WT) compared to non-transgenic (No Tg) mice. n = 4 or 5 mice (7 to 10 eyes) per genotype. **(E)** Hematoxylin and eosin histology photomicrographs showing retinal thickness of the different mouse strains. Cohort of 6-month-old mice, n = 5 to 6 per genotype. **(F)** Morphometry analysis of the total retina, the Outer Nuclear Layer (ONL), and the rest of the retina (Outer Plexiform Layer: OPL, Inner Nuclear Layer: INL, Inner Plexiform Layer: IPL and Retinal Ganglion Cell layer: RGC) of the difference mouse strains at 6 months showing protease-dependent pan-retinal thinning in mice expressing HTRA1 WT (hemi and homo). n= 4 to 6 per genotype. **(G)** Quantification of subretinal Iba1 positive cells present between the RPE and the photoreceptor outer segments (OS) in the retinas of the different mouse strains. n= 4 to 6 mice per genotype, 6-months old, ANOVA test: * $p<0.05$, ** $p<0.01$, *** $p<0.001$, **** $p<0.0001$. **(H)** Western blot of retina lysates from non-transgenic (No Tg) mice or mice transgenic for catalytic dead HTRA1 (S328A, homozygous) or catalytic active HTRA1 (WT, hemizygous or homozygous) incubated with a HTRA1 specific activity-based probe.

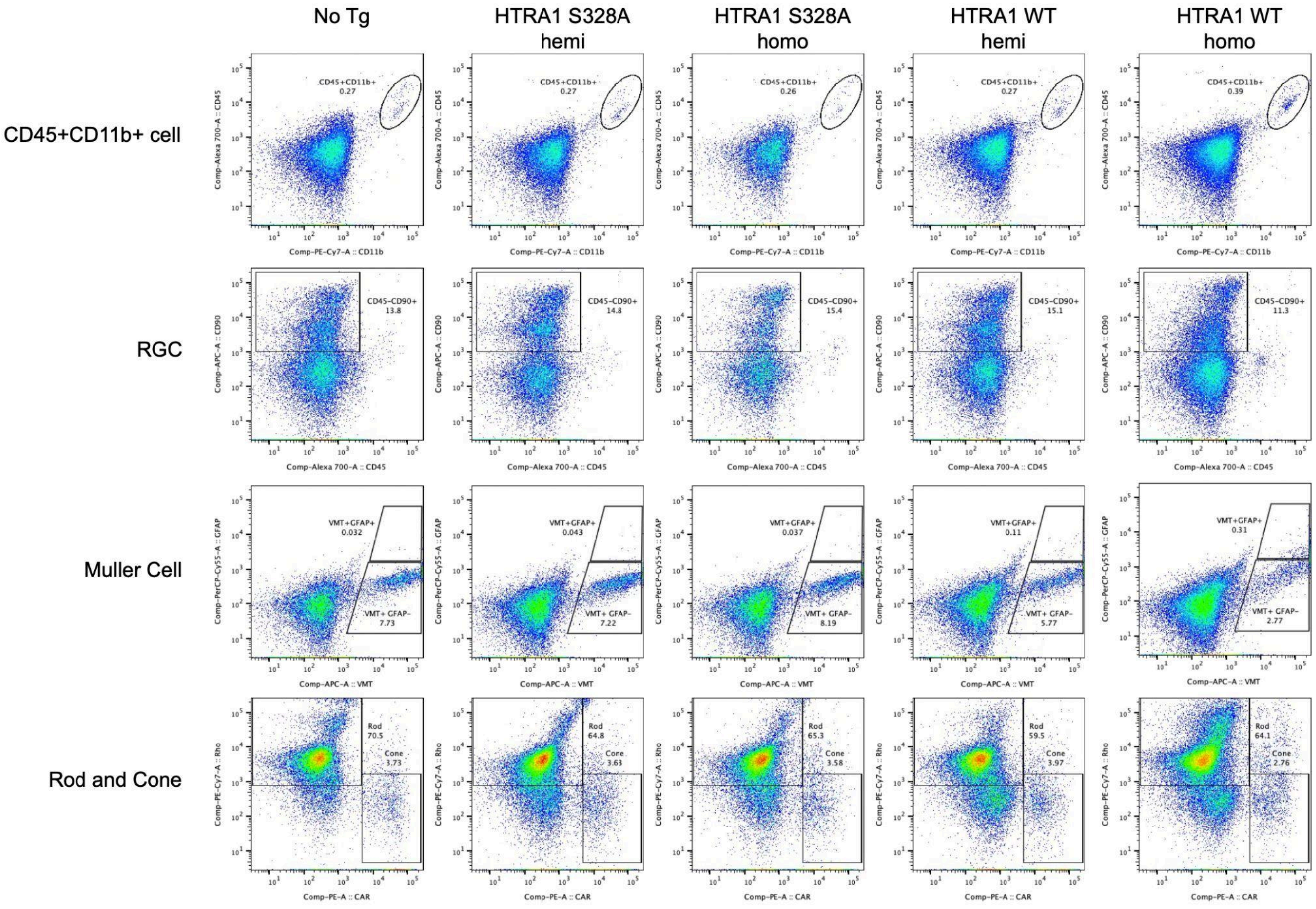

Fig. S2. Representative flow cytometry data used for Fig. 3.

VMT: Vimentin, GFAP: Glial fibrillary acidic protein, Rho: Rhodopsin, CAR: Cone arrestin

Disease Models & Mechanisms • Supplementary information

Table S1. HTRA1 immunoprecipitation from mouse retinal lysates identified 2313 proteins.

Available for download at
https://journals.biologists.com/dmm/article-lookup/doi/10.1242/dmm.052253#supplementary-data

Table S2. SAINT analysis of the comparison between interacting partners of HTRA1 S328A and No Tg identified 37 significant interacting proteins for HTRA1 S328A.

Available for download at
https://journals.biologists.com/dmm/article-lookup/doi/10.1242/dmm.052253#supplementary-data

Table S3. SAINT analysis of the comparison between interacting partners of HTRA1 WT and No Tg identified 36 significant interacting proteins for HTRA1 WT.

Available for download at
https://journals.biologists.com/dmm/article-lookup/doi/10.1242/dmm.052253#supplementary-data
